# Sequential gene expression analysis of myelodysplastic syndrome transformation identifies HOXB3 and HOXB7 as the novel targets for mesenchymal cells in disease

**DOI:** 10.1186/s12885-024-11859-w

**Published:** 2024-01-22

**Authors:** Chunlai Yin, Yanqi Li, Cheng Zhang, Shizhu Zang, Zilong Wang, Xue Yan, Tonghui Ma, Xia Li, Weiping Li

**Affiliations:** 1 Department of Immunology, College of Basic Medical Science, Dalian Medical University, Dalian, Liaoning 116044 China; 2 Department of Hematology, the Second Hospital of Dalian Medical University, Dalian, Liaoning 116027 China

**Keywords:** Myelodysplastic syndromes, Mesenchymal stem cells, Gene expression profiling, Targets, HOXB3, HOXB7

## Abstract

**Background:**

Myelodysplastic syndrome (MDS) is known to arise through the pathogenic bone marrow mesenchymal stem cells (MSC) by interacting with hematopoietic stem cells (HSC). However, due to the strong heterogeneity of MDS patients, it is difficult to find common targets in studies with limited sample sizes. This study aimed to describe sequential molecular changes and identify biomarkers in MSC of MDS transformation.

**Methods:**

Multidimensional data from three publicly available microarray and TCGA datasets were analyzed. MDS-MSC was further isolated and cultured in vitro to determine the potential diagnostic and prognostic value of the identified biomarkers.

**Results:**

We demonstrated that normal MSCs presented greater molecular homogeneity than MDS-MSC. Biological process (embryonic skeletal system morphogenesis and angiogenesis) and pathways (p53 and MAPK) were enriched according to the differential gene expression. Furthermore, we identified HOXB3 and HOXB7 as potential causative genes gradually upregulated during the normal-MDS-AML transition. Blocking the HOXB3 and HOXB7 in MSCs could enhance the cell proliferation and differentiation, inhibit cell apoptosis and restore the function that supports hematopoietic differentiation in HSCs.

**Conclusion:**

Our comprehensive study of gene expression profiling has identified dysregulated genes and biological processes in MSCs during MDS. HOXB3 and HOXB7 are proposed as novel surrogate targets for therapeutic and diagnostic applications in MDS.

## Introduction

MDS is a group of clonal disorders characterized by morphologic dysplasia, ineffective hematopoiesis, and peripheral cytopenia [1], with a high risk of developing acute myeloid leukemia (AML) [2, 3]. The incidence rate of MDS in the general population is 4.5 per 100,000 people per year, but it is higher in males than females (6.2 vs. 3.3 per 100,000 people per year) and substantially increases with age. In addition to gender and age, other risk factors such as chemotherapy drugs, radiation therapy, long-term workplace exposure to benzene, or familial forms can also induce MDS to different degrees [4]. In clinical practice, epigenetic therapy is the main drug that can reverse the repressive state of DNA hypermethylation to relieve symptom, including azacitidine, decitabine, and lenalidomide [5], but sometimes it is still impossible to achieve a proper response to the therapy. Allogeneic HSC transplantation remains the only potentially curative option, but it is so strict that many people cannot be effectively cured through this approach [6]. Some studies indicate that the limited success of HSC transplantation was attributed to the altered BM microenvironment in MDS patients [7].

As one of the main cellular components of the BM microenvironment, MSCs that characterized by the expression of CD73 and CD90 are a group of stem cells that can repair themselves and have the ability to form bone, adipose and nerve cells [8–10]. Functionally, these cells can contribute to reprograming the BM microenvironment by dysregulating the proinflammatory cytokines and inducing the hypoxia, leading to abnormalities in supportive hematopoietic niches [11]. Although some reports suggest that there are no differences in phenotype and growth characteristics of MSC between MDS patients and healthy donors (HD) [12, 13], others believe that even though in the same phenotype MSC can also induce MDS appearing as different characteristics in diverse incubation circumstance [14, 15]. The controversy indicated that there must be some differences in the secreted molecules or cellular effects of MSC which are closely related to the gene expression profile of the MDS, playing an important role in the progression of MDS. However, the mechanism of MSC promoting the progression of MDS needs to be further explored.

Recently, gene chip technology has developed rapidly and been widely used in gene detection. Through it, we have learned that the pathogenesis and progression of heterogeneity in MDS are closely related to the MSC genetic landscape. For instance, Kim Me et al. have reported that MSC could regulate MDS pathogenesis through inflammation and immune dysregulation responses that involve the interferon signaling pathway [16], inducing an immune-suppressive microenvironment in MDS by an indirect mechanism involving monocytes or abnormal transforming growth factor β1, a relevant trigger causing MDS to progress to AML [17]. All of these have shown the feasibility and reliability of exploring MDS-MSC from the perspective of bioinformation. Therefore, a better understanding of the gene expression, developing a comprehensive list, or more consistent testing may help us acquire more useful information to improve the management of patients with MDS.

Driven by the need for effective biomarkers to improve the diagnosis and treatment of MDS, we specifically focused on screening persistently altered genes involved in MDS-MSC. We discovered the novel function of HOXB3 and HOXB7 where gene overexpression is closely associated with MDS progression. Simultaneously, blocking these genes can repair cell proliferation, differentiation, apoptosis and the ability of cells to promote HSC hematopoietic differentiation. Our work identifies HOXB3 and HOXB7 as potential targets for future interventions in MDS.

## Materials and methods

### Study population

Seventeen MDS patients and two healthy controls (They were diagnosed with nonhematologic diseases) were enrolled in this study. Experiments were approved by the ethics committee of the Second Hospital of Dalian Medical University. All study subjects signed a written informed consent before participating in the study.

### RNA information acquisition

The gene expression data of MSC was obtained from the GEO database (**GSE140101, GSE107490 and GSE61853)** (http://www.ncbi.nlm.nih.gov/geo/). All the datasets included healthy donors as control and patients diagnosed with MDS. However, we didn’t analyze the differences in gene expression profiles of BM MSC between MDS subtypes. Total RNA was isolated from BM MSC for gene expression analysis comparing MDS vs. control. Databases were drawn through their portal for analysis [15–17]. The data sets which include the HD and MDS groups were screened and severally analyzed based on GPL10558 (Illumina HumanHT-12 V4.0 expression beadchip), GPL11154 (Illumina HiSeq 2000) and GPL16791 (Illumina HiSeq 2500). Meanwhile, the two samples with missing results in GSE61853 HD group were removed.

### Data processing

The raw data downloaded from GEO were used for further analysis. Data processing mainly utilized a set of different R packages (R version 4.1.0 (2021-05-18)) in Rstudio. We downloaded Gene expression file GSE107490_all_count.txt.gz, GSE140101_ FPKM_GEO.txt.gz, GSE61853_non-normalized.txt.gz and their corresponding annotation platforms from the GEO database. The quality control of each data set was performed to minimize false detection rate (FDR) in original studies by using fastqcr package. Next, the expression matrix was normalized using the normalize Between Arrays function under limma package (version: 3.48.0). Gibberish was then removed. PCA under FactoMineR package was used to verify difference between samples and Reliability of data processing. The normalized data were further processed using the limma package to obtain differentially expressed genes (DEGs) between HD and MDS in these three gene sets. In our study, genes with a p-value less than 0.05 and fold-change greater than 2 were considered as DEGs. Each GSE has been analyzed statistically. Venn diagram tool (http://bioinformatics.psb.ugent.be/webtools/Venn/) was used to help us find overlapped genes.

### GO enrichment and KEGG pathway analysis

To identify the overall overlapped genes enrichment differences between the patients and the controls, we used the Database for Annotation, Visualization and Integrated Discovery (https://david.ncifcrf.gov/, DAVID, version: 6.8) for the further functional enrichment analysis. This website can perform GO and KEGG analyses. The predicted BP (biological process), CC (cell composition), MF (molecular function) of the DEGs were analyzed. Furthermore, the pathways in which the DEGs participated were predicted and mapped using the KEGG database with DAVID. Visualization of the enriched GO terms and KEGG terms were conducted using the GO plot package (version: 1.0.2) in R studio. Terms with p values greater than 0.05 were considered statistically enriched.

### PPI network establishment and further module analysis

To investigate protein-protein interaction function, the Search Tool for the Retrieval of Interacting Genes (http://string-db.org/, STRING) online database was used to identify interactions between known genes and predicted genes at the protein level. Briefly, we input the overlapped genes in the website using the default condition and downloaded the file about these proteins’ interaction to input into Cytoscape software (3.8.2) to obtain gene clusters. The plugins CytoHubba and MCODE in Cytoscape were applied to identify the significant modules in the PPI networks and calculate the degree exhibited by every protein node. Additionally, we extracted PPI pairs based on the combined score over 0.4. The degree cutoff was set to 2, node score cutoff to 0.2, k-score to 2 and Max depth to100 in MCODE.

### Hub genes selection and correlation analysis

The screening of hub genes was mainly conducted through the MCODE plugin of Cytoscape. To perform the correlation analysis between the Hub genes and those genes that had been shown to influence the process of MDS, correlation analysis was carried out using the “tidyr”, “dplyr”, “ggstatsplot”, package (3.14.3) in R. Briefly, selected genes’ expression matrix was imported. The Cor.test function was then executed with the default parameters (type="spearman”) setting. Gene sets with a p-value less than 0.05 were considered to have significantly correlated relations.

### Real-time quantitative polymerase chain reaction

For quantification of gene expression, RNA was isolated from Ctrl (They were diagnosed with nonhematologic diseases) or patients with MDS using a RNeasy Micro Kit or Rneasy Mini Kit (Qiagen). cDNA was synthesized from 1 µg RNA using Superscript IV Reverse transcription (Thermo Fisher) (37 °C for 15 min, 65 °C for 10 min). Real-time PCR analysis was set up with the SYBR Green qPCR Supermix kit (Invitrogen, Carlsbad, CA) and carried out in the iCycler thermal cycler. β-actin was used for normalization. Data were analyzed by the 2^−ΔΔCT^ method [18]. Each sample was analyzed in triplicate, and the analysis was repeated three times. The primers for target genes were as follows.

Primers for * HOXB3*: forward-5′TGCTGCTGGGAGACTCGTAA 3′.

reverse-5′GCATCCCCTTGCAGCTAAAC 3′,

*HOXB5* forward-5′AACTCTCCCCTCCCC ATC 3′.

reverse-5′GGCACTACCCCACCTCAA 3′,

*HOXB6* forward-5′TCC CCTCCCAATGAGTTC 3′.

reverse-5 GCATAGCCCGA CGAATAGA 3′,

*HOXB7* forward-5′CGTCCCTGCCTACAAATC 3′.

reverse-5′GAAGCAAA CGCACAAGAAG 3′,

*SCF* forward-5′ACCCAATGCGTGGACTATCTG 3′.

reverse-5′GGCGACTCCGTTTAGCTGTT 3′,

*TPO* forward-5′CTTCACTGCCTCAGCCAGAAC 3′.

reverse-5′GAATCCCTGCTGCCACTTCA 3′,

*IGF1* forward-5′CCTCTCAAGAGCCACAAATGC 3′.

reverse-5′TCCAGCAGCCAAGATTCAGA 3′,

*IGFBP2* forward-5′TGACAAGCATGGCCTGTACAA 3′.

reverse-5′CACGCTGCCCGTTCAGA 3′,

*CXCL12 *forward-5′ATGTCGAAGCCCCATAGTGAA 3′.

reverse-5′TGGGTGGTGAATCAATGTCCA 3′,

*β-ACTIN* forward-5′CATGTACGTTGCTATCCAGGC 3′.

reverse-5′CTCCTTAATGTCACGCACGAT 3′.

### Cell transfection

MSC cells were seeded 2 × 10^5^ cells per well in 6-well culture plates with DMEM/F-12 medium containing 10% FBS for one day. When cells were grown to a concentration of 70%, transient transfection was performed using the transfection reagent GP-transfectMate (GenePharma, China) according to the manufacturer’s protocol. The MSC were then transfected with HOXB3 or HOXB7 small interfering RNA (siRNA) (GenePharma, China) and a non-speciffc control siRNA (NCsiRNA). SiRNA was mixed with GP-transfect-Mate transfection reagent in serum-free medium according to the manufacturer’s instructions incubated for 6 h, then the cells were incubated in a Growth Medium for following analysis. The siRNA sequences are as follow:

siRNA HOXB3: sense-5′GAUGAAAG AGUCGAGGCAATT 3′

antisense-5′UUGCCUCGACUCUUUC AUCTT 3′,

siRNA HOXB7: sense-5′GCUAUUGUAAGGUCUUUGUTT 3′

antisense-5′ACAAAGACCUUACAAUAGCTT 3′

### CCK-8 assay

MSCs were seeded in 96-well cell culture plates. Cells were transfected when they reached a density of 40–60%. Cell proliferation was assessed every 24 h after transfection by measuring absorbance at 450 nm with a multiskan-FC (Thermo Fisher) according to the instructions of the CCK-8 kit (Vazyme).

### apoptosis assay

After 24 h of transfection, the cells were treated according to the instructions of the eBioscience Annexin V-FITC Apop Kit (Thermo Fisher) and detected by flow cytometry. Finally, the data were statistically analyzed using Graphpad Prism software to plot the cell distribution. Three replicates were set for each group.

### Assay of adipogenic differentiation and osteogenic differentiation

After transfecting with HOXB3 or HOXB7, MSC were collected and cultured with adipogenesis induction medium (α-MEM containing 10% FBS, 5 µg/mL insulin, 0.5 mmol/L 3-isobutyl-1-methylxanthine, and 1 µmol/L dexamethasone) in 6-well plates with 1 × 10^5^ cells per well for 14 days. Every three days, we changed culture medium. We used Oil Red O staining to distinguish mature adipocytes from preadipocyte during the process of culture. For osteogenic differentiation, MSC were inoculated in 6-well plates with 1 × 10^5^ cells per well and cultured in freshly formed osteogenic medium (OM) for 21 days. Alizarin Red staining was used to detect bone mineralization.

### Colony forming unit (CFU) assay

MDS-MSC (with or without HOXB3/7 treatment) were plated at a near confluent density of 1.0 × 10^4^ cells per well in 48-well plates. 24 h later, healthy CD34 ^+^ HSC were seeded in contact with the MSC feeder layer at a density of 5.0 × 10^5^ cells per well in hematopoietic media and cultured for up to 5 days. Then, HSC were cultured in methylcellulose media (MethoCult™ H4434, STEMCELL) for 14 days and colonies were counted using an inverted microscope.

### Survival analysis

The overall survival (OS) analysis of hub genes was conducted using the Kaplan-Meier curve in cBioPortal. cBioPortal (http://www.cbioportal.org) is an online analysis platform for multidimensional cancer genomic data that visualizes genes, samples and data types. Survival analysis was performed by alternating the Hub genes on line. And P-value less than 0.05 was considered statistically significant.

### Statistical analysis

The data are displayed as histograms and line charts. The parameters were compared using one- or two- way analysis of variance. Data are representative of at least three independent experiments. Statistical analysis was performed using Prism 8.4.3 (GraphPad Software, La Jolla, California). A p-value lower than 0.05 was considered statistically significant.

## Results

### Data series and DEGs identification

**Fig. 1:**
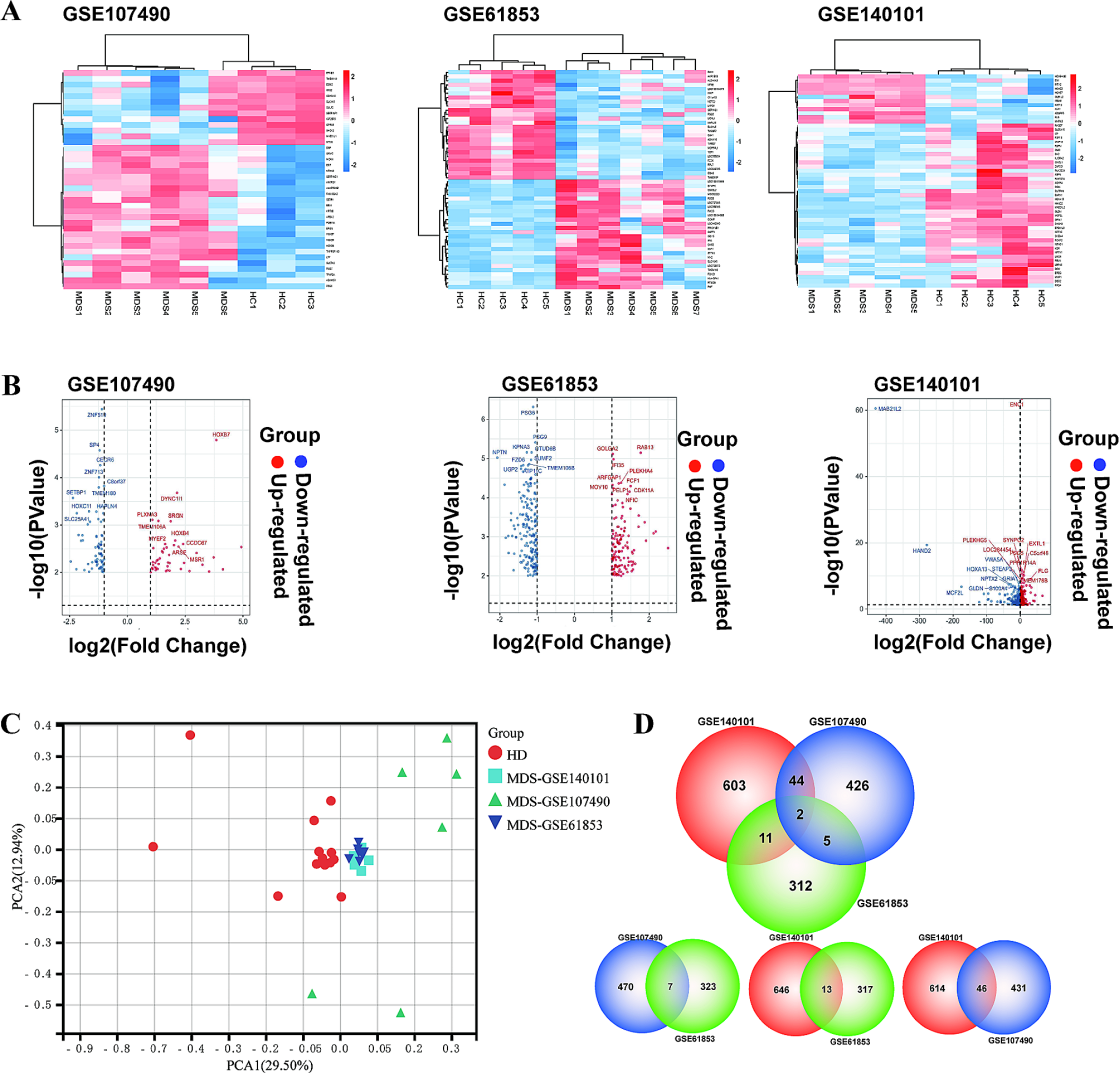
Heatmaps, Volcano plots of the DEGs in three datasets and the intersections of DEGs between groups. (**A**) Heatmap of the dysregulated RNA expression profiles in HD and MDS patients. 477 DEGs in GSE170490, 330 DEGs in GSE61853 and 660 DEGs in GSE140101. (**B**) Volcano plots of the DEGs in GSE140101, GSE107490 and GSE16853 cohorts. Red points: significantly upregulated DEGs, blue points: significantly downregulated DEGs. (**C**)PCA of RNA-seq data from HD-MSC and MDS-MSC (*n* = 13 and *n* = 18, respectively), Each symbol represents one sample. (**D**) Venn diagram of the DEGs in MDS/HD among GSE107490, GSE61853 and GSE140101 and the DEGs in MDS/HD between DEGs of different 2 groups in the 3 GSE

We obtained the gene expression profiles from three data series (**GSE61853, GSE140101**, and **GSE107490**) of MSC samples in BM from MDS patients and HD in the GEO database. We picked and analyzed the corresponding samples in series matrix by Rstudio (the details were shown in the Data Sheet). As shown in Fig. 1, we screened 330, 660 and 477 differentially expressed genes from GSE61853, GSE140101 and GSE107490, respectively, based on the criteria of P values less than 0.01 and| log2FC| over 1.0, and we displayed the top 13% of total DEGs (Fig. 1A). In addition, we created volcano plots for the three data series to present the top 10 up-regulated and down-regulated genes from different data series (Fig. 1B). PCA demonstrated reliability of data processing of these three-date series is credible (Fig. 1C). Next, we obtained the overlap among each two DEG profiles via Venn analysis. The results showed that 62 DGEs were captured totally by intersections (Fig. 1D). Notably, two DEGs showed obvious differential expression in all these groups, including *PSG5* (the human pregnancy-specific glycoproteins) and *SLC5A3* (solute carrier family 5 - inositol transporters member 3) (Fig. 1D). Furthermore, all of 54 of 62 shared genes exhibited the same tendency, and the hub genes were presented in Table 1.

**Table 1:** 62 shared DGEs in GSE61853、GSE140101、GSE107490

Gene Symbol	Log2FC	P-value	Gene Set
ALDH1B1	2.21959644	2.36E-05	GSE140101
	0.863379065	0.344701164	GSE107490
	1.41306868	2.28E-03	GSE61853
ANKRD1	4.275054878	3.67E-05	GSE140101
	4.64720788	0.029697897	GSE107490
B3GALT2	4.640003443	0.025740299	GSE140101
	1.778463016	0.04390112	GSE107490
BDNF	2.751651406	2.63E-05	GSE140101
	1.58090477	0.003192248	GSE107490
C15orf54	7.079804298	0.00041988	GSE140101
	2.459431618	0.022730368	GSE107490
C8orf34	4.604160965	0.00035933	GSE140101
	2.510961919	0.020938048	GSE107490
CAMK2N1	-5.714347351	0.00211602	GSE140101
	-1.867105729	0.245696484	GSE107490
	-1.24800022	9.96E-03	GSE61853
CD9	-3.232786976	0.00013667	GSE140101
	-1.73470962	0.410107417	GSE107490
	1.04047878	9.19E-03	GSE61853
CDH6	4.863804133	0.005834862	GSE140101
	1.62250771	0.109191604	GSE107490
	1.36463978	4.02E-03	GSE61853
DNAJB4	2.546720106	4.22E-06	GSE140101
	1.109473261	0.034613621	GSE107490
DYNC1I1	2.876588418	0.001333049	GSE140101
	2.146208933	0.00020978	GSE107490
EAPP	-1.040370231	0.041181242	GSE107490
	1.11049122	6.00E-03	GSE61853
EGF	3.116701167	0.000348036	GSE140101
	2.259941208	0.030368795	GSE107490
ENC1	5.027114058	0	GSE140101
	2.046657231	0.045898614	GSE107490
EPGN	9.828095173	7.58E-06	GSE140101
	4.236634344	0.039756196	GSE107490
ERCC6	2.654053664	0.000253386	GSE140101
	1.275862646	0.005634787	GSE107490
FAM198B	2.123823258	0.00706219	GSE140101
	1.18243362	1.40E-03	GSE61853
FAM212B	3.456298789	4.92E-05	GSE140101
	1.50250034	0.046021972	GSE107490
FZD7	1.253385455	0.049964129	GSE107490
	1.00383188	5.28E-03	GSE61853
GADD45A	2.005328331	0.000937253	GSE140101
	0.99970494	0.0162257	GSE107490
GPR88	-8.422226184	0.003730458	GSE140101
	-3.714245517	0.029006806	GSE107490
GRIK2	7.905879415	2.88E-05	GSE140101
	3.011227255	0.025362395	GSE107490
HGD	13.58892984	2.72E-05	GSE140101
	2.452858965	0.006307949	GSE107490
HOXB3	5.236159051	0.016893139	GSE140101
	1.579025476	0.010187243	GSE107490
HOXB6	8.105207982	0.000483471	GSE140101
	4.932214752	0.002929186	GSE107490
HOXB7	7.759393231	0.003021712	GSE140101
	3.853829352	1.61E-05	GSE107490
INTU	2.583050627	0.001305576	GSE140101
	1.151648631	0.012732716	GSE107490
ITIH3	15.50133211	0.001053709	GSE140101
	2.042310805	0.046335147	GSE107490
KCNJ15	-2.13961095	0.043310163	GSE140101
	-1.430569355	0.04089291	GSE107490
KCNK6	2.372837201	0.000899832	GSE140101
	1.282018493	0.016508652	GSE107490
KRT18	6.898239866	0.039784339	GSE140101
	2.014370575	0.029440096	GSE107490
LIF	3.791257105	1.19E-07	GSE140101
	2.037364823	0.025122453	GSE107490
LIME1	2.364949856	0.00866422	GSE140101
	-1.36085712	1.69E-03	GSE61853
LRP2	5.996437844	0.040237403	GSE140101
	2.04439412	0.046088593	GSE107490
MAPK8IP3	2.377091945	1.59E-06	GSE140101
	-1.55323374	2.65E-03	GSE61853
MCAM	4.58613714	8.22E-06	GSE140101
	2.23147175	0.048866356	GSE107490
MT1M	-4.629553487	0.000288173	GSE140101
	-2.110806786	0.00162601	GSE107490
NCKAP5	2.28353833	0.047374356	GSE140101
	1.711999999	0.01646364	GSE107490
NEDD9	2.077137188	0.027244062	GSE140101
	1.01537145	1.19E-03	GSE61853
NRXN3	4.087492066	0.034205593	GSE140101
	5.140588996	0.012456264	GSE107490
P4HA2	1.378157612	0.016172506	GSE107490
	1.00517785	7.08E-03	GSE61853
PDE11A	7.087082917	3.06E-05	GSE140101
	2.670760374	0.04506532	GSE107490
PIDD1	2.422051168	0.000613631	GSE140101
	-1.25465901	3.36E-04	GSE61853
PITX2	12.26269235	2.26E-06	GSE140101
	1.624332995	0.036100387	GSE107490
PLOD2	2.236686901	0.000899832	GSE140101
	1.34334538	0.03777175	GSE107490
PSG5	5.656918278	3.25E-11	GSE140101
	2.812986404	0.014281341	GSE107490
	1.83988636	1.06E-03	GSE61853
PSG9	3.733354341	0.00482393	GSE107490
	1.04989238	3.88E-06	GSE61853
RIMS1	2.293992579	0.012139472	GSE140101
	2.016059851	0.029145619	GSE107490
RPL7	-1.212806431	0.027409272	GSE107490
	1.43503758	1.02E-04	GSE61853
SALL2	-2.381052934	0.020669261	GSE140101
	-1.371240858	0.004520103	GSE107490
SERPINE1	2.613100547	0.01296588	GSE140101
	2.690383234	0.00941362	GSE107490
SH3D21	4.489910142	0.000247259	GSE140101
	1.189154473	0.044128777	GSE107490
SHOX2	-7.192959424	0.000375349	GSE140101
	-4.109489148	0.035336319	GSE107490
SLC5A3	-3.530470591	0.00510518	GSE140101
	-1.54904469	0.047566341	GSE107490
	-1.56751485	4.39E-03	GSE61853
SLFNL1	3.751206659	0.003042524	GSE140101
	2.453839771	0.027687351	GSE107490
SYT7	-3.648470882	0.023096348	GSE140101
	-2.551726171	0.021606244	GSE107490
TBX15	-2.079643894	0.000212731	GSE140101
	-1.939126001	0.001648641	GSE107490
TFAP2A	11.59783353	0.004329744	GSE140101
	3.701448243	0.046375614	GSE107490
TMEM119	-2.141554705	0.016963893	GSE140101
	-2.065941723	0.018775964	GSE107490
WASH3P	2.639155175	0.005297574	GSE140101
	-1.19195718	2.44E-03	GSE61853
ZNF692	2.184216641	0.00041791	GSE140101
	-1.10851553	8.13E-04	GSE61853

### The key pathways and PPI network analyses of DEGs

**Fig. 2:**
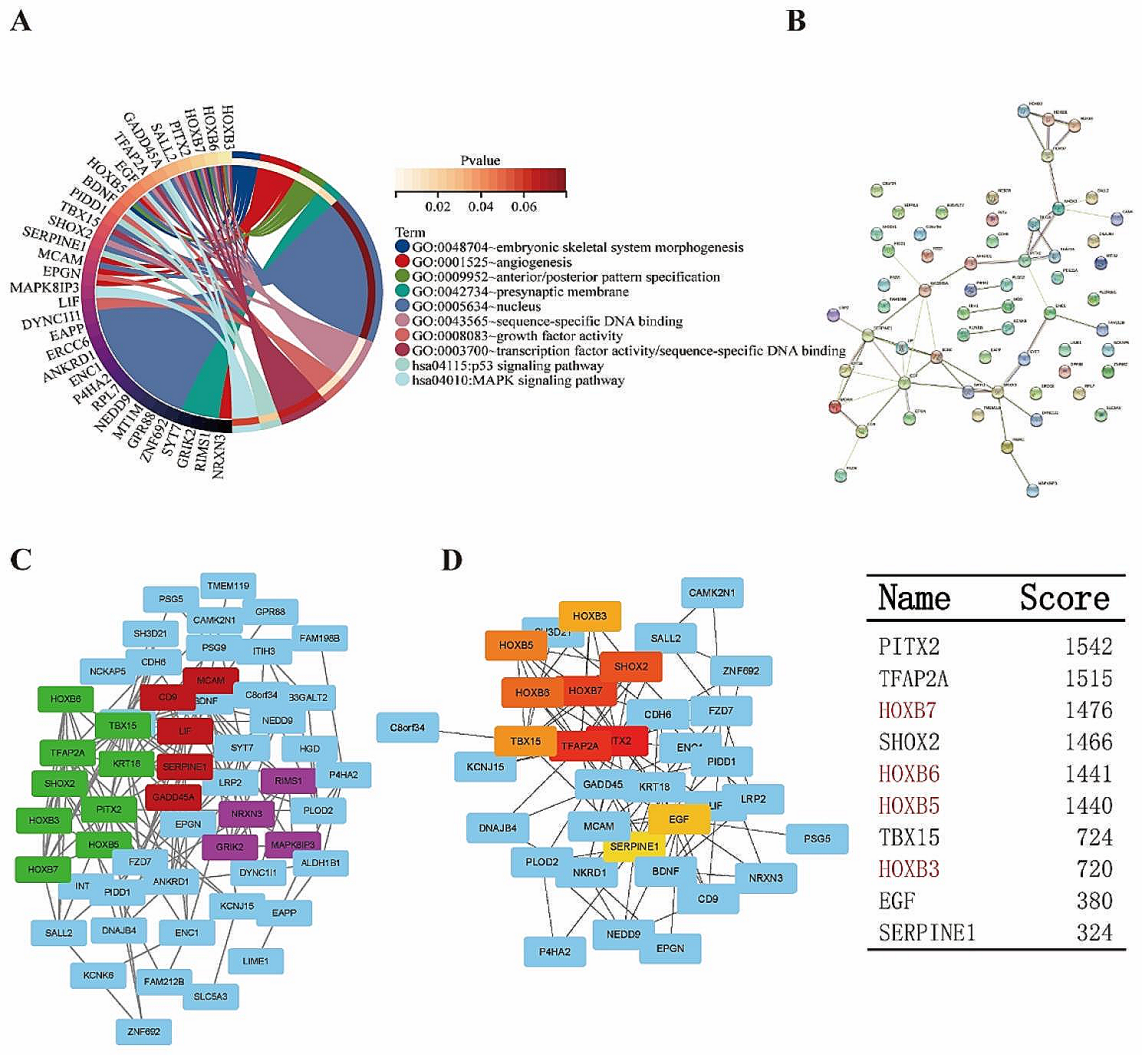
DAVID analysis and PPI enrichment of the 62 shared genes. (**A**) Functional enrichment analysis of 62 shared genes identified in MSC from MDS patients through GO terms and KEGG pathway with DAVID analysis. The Top 10 was shown in the figure. (**B**) The analysis of Protein-Protein Interaction Networks for the 62 genes. (**C**) Green: cluster 1: Interaction was the most obvious shown by MCODE in Cytoscape. Red: cluster 2: The second cluster. Purple: cluster 3: The third cluster. (**D**) Hub Gene was found by cytohubba in Cytoscape. The score of hub genes is shown in the table

To gain further insight into the function of the 62 shared DEGs, we performed functional and pathway enrichment analyses using DAVID. The top 12 results were showed in Table 2. The functions were categorized into 4 groups: BP, CC, MF and KEGG pathway. GO analysis revealed that the function of DEGs mainly enriched in following term, embryonic skeletal system morphogenesis, angiogenesis, anterior/posterior pattern specification, sequence-specific DNA binding, platelet degranulation, growth factor activity, presynaptic membrane, negative regulation of transcription from RNA polymerase II promoter, positive regulation of epidermal growth factor-activated receptor activity, regulation of protein localization to cell surface, anatomical structure morphogenesis. KEGG pathway mainly showed enrichment in p53 signaling pathway and MAPK signaling pathway (Fig. 2A).

**Table 2:** Top 12 GO terms and KEGG pathways associated with the 62 genes identified via DAVID analysis

Term	Count	P-value	Genes
embryonic skeletal system morphogenesis	4	2.65E-04	HOXB3, HOXB7, HOXB6, HOXB5
angiogenesis	6	7.36E-04	EPGN, EGF, MCAM, SERPINE1, NRXN3, HOXB3
anterior/posterior pattern specification	4	0.002168409	HOXB3, HOXB7, HOXB6, HOXB5
sequence-specific DNA binding	7	0.004101735	TFAP2A, SALL2, SHOX2, HOXB3, PITX2, HOXB7, HOXB6
platelet degranulation	4	0.004431234	ITIH3, EGF, SERPINE1, CD9
growth factor activity	4	0.012227549	EPGN, BDNF, EGF, LIF
presynaptic membrane	3	0.015587105	RIMS1, GRIK2, SYT7
p53 signaling pathway	3	0.022469123	PIDD1, GADD45A, SERPINE1
negative regulation of transcription from RNA polymerase II promoter	7	0.027411597	TFAP2A, TBX15, SALL2, SHOX2, ANKRD1, HOXB3, PITX2
regulation of protein localization to cell surface	2	0.028579562	BDNF, EGF
positive regulation of epidermal growth factor-activated receptor activity	2	0.028579562	EPGN, EGF
anatomical structure morphogenesis	3	0.03534194	KRT18, MCAM, HOXB5

To analyze the interaction among the DEGs, we used STRING to assist in determining the PPI network. The results showed that the PPI network involved 62 nodes together with 132 edges (Fig. 2B). Furthermore, we obtained three significant modules based on the degree of importance by utilizing cluster analysis of the PPI network in Cytoscape MCODE. Module 1 contained 9 nodes and 81 edges; Module 2 contained 5 nodes and 23 edges; Module 3 contained 4 nodes and 16 edges (Fig. 2C). The top cluster included *KRTI8*, *TFAP2A*, *PITX2*, *HOXB5*, *SHOX2*, *TBX15*, *HOXB3*, *HOXB6*, and* HOXB7*. Then, we identified the top ten genes under the evaluation of the degree of connectivity in PPI network. These results indicated that *PITX2* (score = 1542) exhibited the largest score of connectivity, followed by *TFAP2A* (score = 1515), *HOXB7* (score = 1476), *SHOX2* (score = 1466), *HOXB6* (score = 1441), *HOXB5* (score = 1440), *TBX15* (score = 724), *HOXB3* (score = 720), *EGF* (score = 380), *SERPINE1* (score = 324) (Fig. 2D). Based on the results of DAVID clustering and degree score, we focused on possible potential core genes HOX (homeotic genes) and its four subtypes: HOXB3, HOXB5, HOXB6, HOXB7.

### Blocking HOXB3 or HOXB7 could repair the function of MSC

**Fig. 3:**
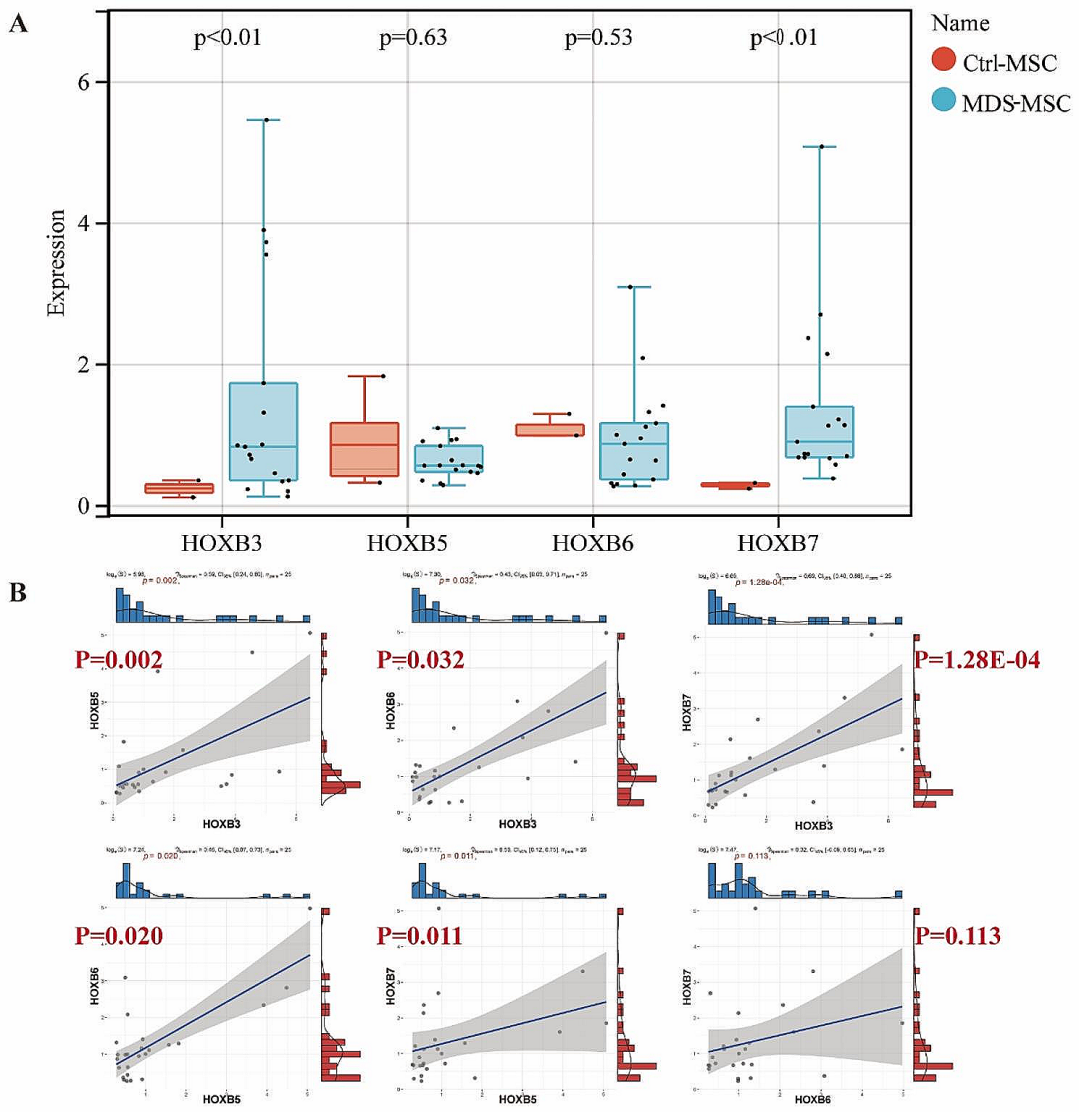
Quantitative PCR validated *HOXB3*, *HOXB7* expression increased in MDC-MSCs and correlation analysis between core genes. Total RNA was isolated from HD-MSCs, MDS-MSC, and the total mRNA was reverse transcribed into cDNA and analyzed by real-time quantitative PCR for (**A**). (**B**) Correlation analysis between core genes

Over all, we found that the expression of *HOXB3*, *HOXB5*, *HOXB6*, HOXB in MDS-MSC was significantly upregulated. To confirm the expression changes founded in the whole genome sequencing, we performed qRT-PCR to verify results of *HOXB3*, *HOXB5*, *HOXB6* and *HOXB7* DGE in MDS-MSCversus HD-MSCs. As expected, *HOXB3* and *HOXB7* were highly expressed in MDS-MSC compared with Ctrl-MSCs. But we didn’t obtain the difference of *HOXB5* and *HOXB6* at the transcript level because of the limited sample size (Fig. 3A). Subsequently, we performed correlation analysis between *HOXB3*, *HOXB5*, *HOXB6* and *HOXB7* in sample data and found that the expression of hub genes was correlated with each other (Fig. 3B). Based on the bioinformatics results, we selected *HOXB3* and *HOXB7* as the next key research objects.

**Fig. 4:**
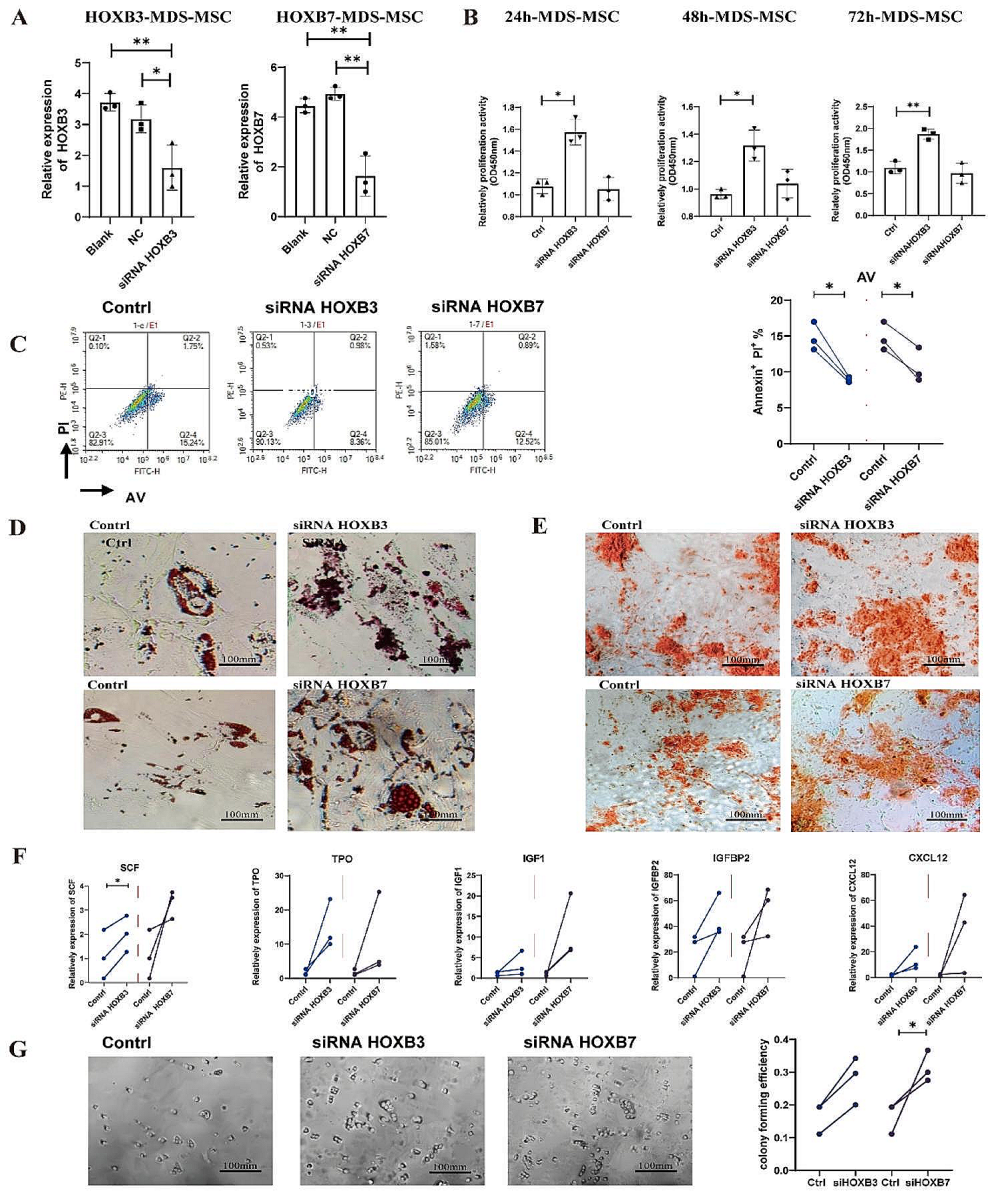
Cell function analysis after blocking HOXB3 and HOXB7. (**A**) Interference efficiency of siRNA after cultured 48 h. (**B**) CCK-8 method to assess the effect of MSC-HOXB3 and MSC-HOXB7 on the proliferation in MSC. (**C**) Cell apoptosis analyzed after downregulating the expression of HOXB3 and HOXB7 by flow cytometry. (**D**) Oil Red O staining analyzed adipogenic differentiation after downregulating HOXB3 and HOXB7 MDS-MSC. (**E**) Alizarin Red staining analyzed osteogenic differentiation after downregulating HOXB3 and HOXB7 MDS-MSC. (**F**) The expression of hematopoiesis-related genes in MSC were analyzed by RT-PCR after downregulating *HOXB3* and *HOXB7* 48 h. (**G**) CFU-colony forming of HSC were analyzed after co-incubation with MSC that downregulating the expression of HOXB3 and HOXB7. (* indicates significant difference, *P* < 0.05; scale bar: 100 μm)

To analyze the effect of HOXB3 and HOXB7 on MSCs’ function, cells were transfected with siRNA separately to block the expression of HOXB3 and HOXB7. Subsequently, RT-qPCR was performed to verify the effectiveness of siRNA. The mRNA level of *HOXB3* and *HOXB7* was significantly reduced upon transfecting (Fig. 4A). Cell number is closely related to cell function. First, we performed the CCK-8 assay to analyze cell proliferation. The results showed that HOXB3, but not HOXB7, was effective in promoting the proliferation of MSC, as indicated by the statistically significant difference between the siRNA HOXB3 group and the control group (*P* < 0.05) (Fig. 4B). Then, cell apoptosis was analyzed through flow cytometry. We found that blocking HOXB3 or HOXB7 could reduce MSCs apoptosis (Fig. 4C). As we all know, MSCs can differentiate into different cells to support bone marrow function. The differentiation ability of MSCs was analyzed after blocking HOXB3 or HOXB7. The results showed that blocking HOXB3 or HOXB7 could strengthen the formation of lipid droplets (Fig. 4D), and significantly enhanced mineral deposition compared to the control (Fig. 4E). All these results revealed that HOXB3 and HOXB7, which were overexpressed in MDS-MSC, inhibited cell proliferation, adipogenic differentiation, osteogenic differentiation and promoting cell apoptosis.

It has been reported that MSC secrete some hematopoietic cytokines, which play a role in hematopoietic support functions of MSC [19–21]. In addition to the effects on MSC cells themselves, we also examined the function that supporting hematopoiesis differentiation of HSCs. RT-qPCR assay showed that mRNA expression of *SCF*, *TPO*, *IGF1*, *IGFBP2* and *CXCL12*, which could support HSC function, was increased after blocking HOXB3 or HOXB7 in MSCs. There was a statistically significant difference in expression of SCF between the siRNA HOXB3 group and the control (*P* < 0.05) (Fig. 4F). In addition, HSC had high CFU-colony forming efficiency after co-incubation with MSC that downregulated the expression of HOXB3 or HOXB7 (Fig. 4G). All these results revealed that blocking HOXB3 or HOXB7 in MSCs could repair the function that supporting hematopoiesis differentiation of HSCs.

### The identification of hub genes in MDS progression

**Fig. 5:**
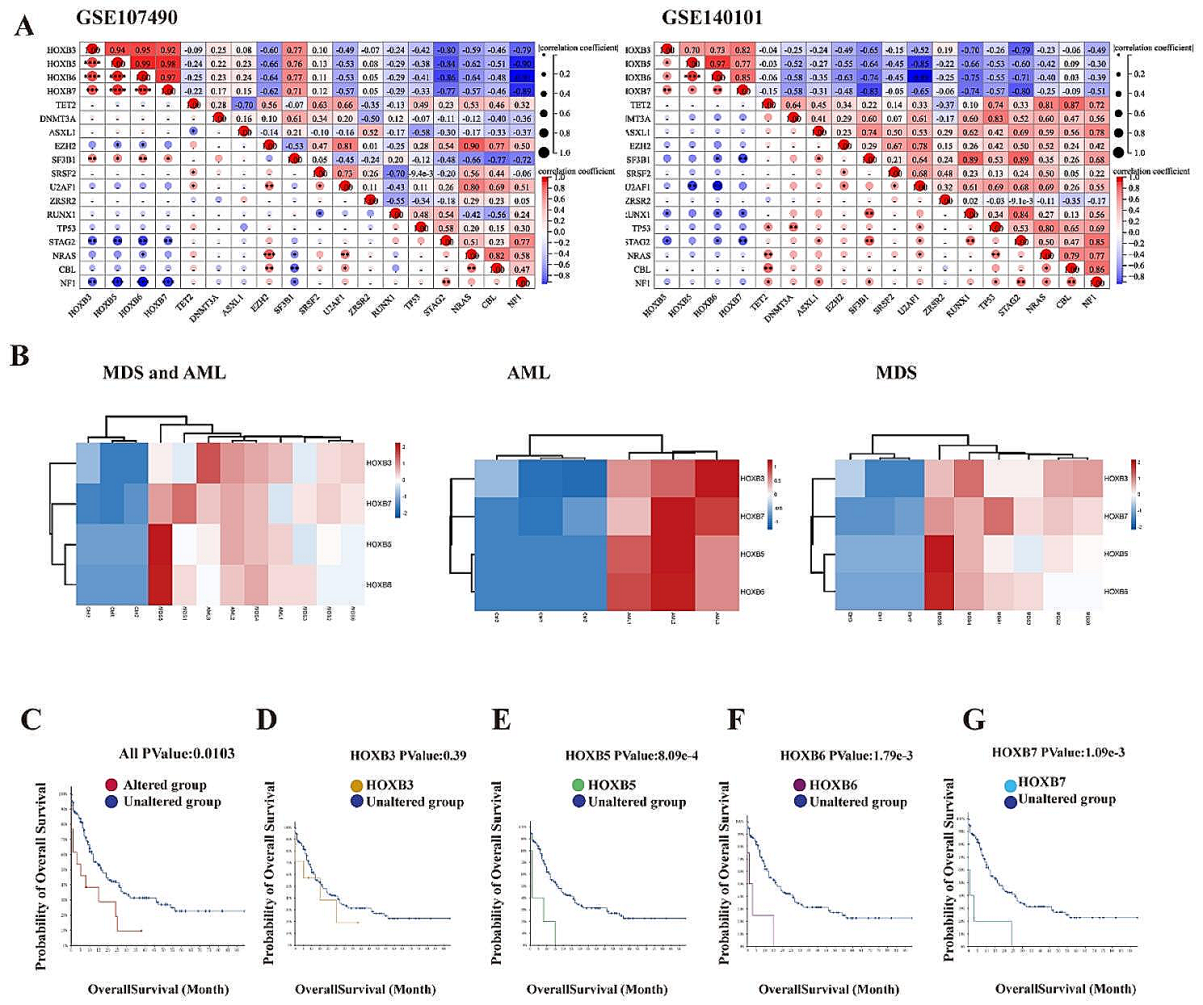
Correlation analysis and survival analysis process involving the core genes. (**A**) Relation between Hub genes mRNA expression levels and mRNA expression at recognized susceptibility genes in MDS from the GSE107490, GSE140101. *P* < 0.05 was considered statistically significant. (**B**) Hub genes HOXB3, HOXB5, HOXB6, HOXB7 expression profile between HD, MDS and AML. Overall survival (OS) difference of normal and altered Hub genes expression groups in significantly prognostically relevant tumors from CBioPortal (http://www.cbioportal.org). *P* < 0.05 was considered statistically significant. (**C**) All the Hub genes; (**D**) HOXB3; (**E**) HOXB5; (**F**) HOXB6; (**G**) HOXB7

To predict the role of hub genes, we screened a series of genes that have been shown to influence the process of MDS, such as *TET2*, *DNMT3A*, *ASXL1*, *EZH2*, *SF3B1*, *SRSF2*, *U2AF1*, *ZRSR2*, *RUNX1*, *TP53*, *STAG2*, *NRAS*, *CBL*, *NF1*, for correlation analysis. The results showed that the hub genes were significantly correlated with disease genes in GSE140101 and GSE107490, respectively (Fig. 5A).

As we all know, MDS high-risk patients have an increased propensity to evolve to AML. Therefore, we first selected the expression matrix of Hub genes in HD, MDS and AML to plot a heatmap (Fig. 5B). The Kaplan-Meier curve was employed to predict the prognosis of the 4 identified Hub genes. Among the genes examined, MDS patients having HOXB3, HOXB5, HOXB6, HOXB7 alterations showed worse Overall Survival (OS) (Fig. 5C). Although the difference of HOXB3 was not statistically significant, the OS of patients having HOXB3 alteration showed an obvious decline tendency (Fig. 5D). Survival analysis showed that HOXB5, HOXB6, HOXB7 alterations significantly shortened survival (Fig. 5E, F and G).

## Discussion

MDS is a clonal hematopoietic system disease that is difficult to diagnose, characterized by reduced hematopoietic function, peripheral blood cytopenia and morphogenesis [22]. According to the revised version of the International Prognostic Scoring System (IPSS-R), MDS can be divided into different subtypes. The treatment for MDS in the risk group included component blood infusion, hematopoietic factor therapy, immunomodulator and epigenetic drug therapy. But only a few drugs are currently available for treatment, more drugs are now under clinical investigation [23], and overcoming MDS remains a challenge for us.

In recent years, targeted therapies are emerging for small subsets of MDS patients with specific somatic mutations (such as TP53, IDH1/2, FLT3). But currently, they have not been approved widely for use as mutation-directed medications of treating MDS [23]. At the same time, accumulated data indicate that MSC in MDS model display aberrant characteristics contributing to disease initiation and transformation into AML [24]. Hence, it is very urgent to identify potential markers, especially in MSC, to promote the diagnosis and prognosis of MDS. Therefore, in the research we analyzed the potential therapeutic targets of MSC in MDS patients based on bioinformatics, to find potential therapeutic targets of MDS.

Due to the strong heterogeneity of MDS patients and significant changes in the course of the disease, it is difficult to find common targets in limited sample size studies. Here, we used three databases containing MDS and HD samples, including **GSE140101, GSE107490**, and **GSE61853** from GEO. Significantly, differences of PSG5 (a putative AF (Amniotic fluid)-MSC markers [25]) and SLC5A3 (essential to support a myo-inositol auxotrophy in AML [26]) were expressed in various stages of MDS in these data. In order to find more potential targets on MDS-MSC, we integrated the differential genes in three databases to obtain 62 differential genes, all of them appear more than twice in three Dataset. Furthermore, GO enrichment analysis indicated that the identified DEGs were mainly enriched in embryonic skeletal system morphogenesis, angiogenesis, anterior/posterior pattern specification, sequence-specific DNA binding and platelet degranulation. They were all related to growth and development, which showed abnormal biological processes associated with cellular phenotypes and transcriptional regulation in MSC. Actually, they had been taken as the more important cause of MDS, similar to the study in 2013 conducted by Geyh S et al., in which they reported that MSC are structurally, epigenetically and functionally altered, which leads to impaired stromal support and seems to contribute to deficient hematopoiesis in MDS [27]. Then KEGG enrichment analysis shown the difference were enriched in P53 signaling pathway representing the tumor suppression and MAPK signaling pathway, playing a key role in the differentiation, proliferation and apoptosis of cells [28, 29]. This clearly proved that inhibition of MSC played important roles in the transformation from MDS to AML, although some research had shown that there were no differences observed with respect to phenotype, differentiation capacity, immunomodulatory capacity or hematopoietic support in MSC between MDS and HD [30].

By establishing the PPI network of DEGs, we picked out HOXB3, HOXB5, HOXB6, and HOXB7 as hub genes with the highest degrees. And, our experiment results manifest that HOXB3 and HOXB7 significantly regulates hematopoiesis capacity in MSC at the process of MDS and plays a key role. Homeotic (HOX) genes, a group of genes regulating the body shape, are developed in regulatory system and transcription factor that causes cell differentiation blocking and malignant self-renewal [31, 32]. The role of the HOXB3 varies in different tumors. Some studies suggested that loss of HOXB3 correlates with the development of hormone receptor negative breast cancer [31], or act as tumor suppressors through FLT3-ITD driver in AML [33]. But some scholars believe that HOXB3 promotes prostate cancer cell progression by transactivating CDCA3 [34]. We found that the gene expression of HOXB3 was increased significantly indicating that it may associate with malignant lesions of MDS. Different from HOXB3, HOXB5 was negatively correlated with myeloid cell differentiation signaling [35], but promoted tumor aggression and progression of various tumors including AML [36, 37]. According to our results, HOXB5 may promote the progression of MDS to AML. Abnormalities of the HOXB6 expression in granulopoiesis and monocytopoiesis may contribute to the development of the leukemic phenotype appearing as its overexpression in murine BM and generating a myelomonocytic precursor in vitro [38], and causes HSC expansion and AML in vivo [39]. As expected, HOXB6 increased significantly also in MDS-MSC in our results. HOXB7 that can induce activation of MAPK/ERK pathway which promotes tumor progression is also upregulated in MDS-MSC [40]. The results implied that the HOX family, especially the overexpression of HOXB3, HOXB5, HOXB6, HOXB7, played important roles in normal and malignant hematopoiesis in MSC of MDS. Furthermore, to demonstrate the importance of hub genes, we screened a series of genes, including TET2, DNMT3A, ASXL1, EZH2, SF3B1, SRSF2, U2AF1, ZRSR2, RUNX1, TP53, STAG2, NRAS, CBL, NF1, which had been shown to influence the procedure of MDS, for correlation analysis. As predicted, significant correlations indicated the important roles of hub genes. At the same time, survival analysis showed that MDS patients having HOXB3, HOXB5, HOXB6, HOXB7 alterations showed worse OS. Furthermore, we explored the function of hub genes in MDS-MSC that obtained through bioinformatics analysis *in vitro.* The results showed that compared with other genes, HOXB3 and HOXB7 could regulate the function of MSCs cells to a greater extent. But because of the size and source of sample, the results need to be more explored.

However, due to a shortfall of the underlying data acquisition technology, false positive results may occur, which is also the biggest flaw of this paper. In addition, recently, different views on the pathogenic role of MSC in MDS have been raised. The researchers believed that although MDS-MSC displayed higher mutational burdens compared to healthy MSCs, no evidence for acquired mutations as disease initiators for MDS was found [41]. Next, more samples of MDS patients will be collected to verify the conclusions through experiments.

In conclusion, our findings predicted that dysplasia of MDS-MSC is closely related to the pathogenesis of MDS through altered HOXB family, providing potential targets for therapeutic and diagnostic applications in MDS.

## Data Availability

The data that support the findings of this study are openly available in National Center for Biotechnology Information at https://www.ncbi.nlm.nih.gov/, with the reference number as GSE140101, GSE107490 and GSE61853. The OS analysis of hub genes was performed using the Kaplan-Meier curve in cBioPortal (http://www.cbioportal.org).

## Abbreviations

MDS: Myelodysplastic syndrome
BM: Bone marrow
MSC: Mesenchymal stem cells
HSC: Hematopoietic stem cells
HD: Healthy donors
AML: Acute myeloid leukemia
BP: Biological process
CC: Cell composition
MF: Molecular function
FDR: False detection rate
GO: Gene Ontology
KEGG: Kyoto Encyclopedia of Genes and Genomes
OS: Overall Survival
